# Evaluation of rat liver with ARFI elastography: *In vivo* and *ex vivo* study

**DOI:** 10.1371/journal.pone.0217297

**Published:** 2019-05-23

**Authors:** Guillermo Carbonell, Juan de Dios Berná-Serna, Lidia Oltra, Carlos M. Martínez, Nuria Garcia-Carrillo, Florentina Guzmán-Aroca, Francisco Javier Salazar, José Tudela, Juan de Dios Berná-Mestre

**Affiliations:** 1 Department of Radiology, Virgen de la Arrixaca University Clinical Hospital, University of Murcia, Murcia, Spain; 2 Institute of Biomedical Research (IMIB), Virgen de la Arrixaca University Clinical Hospital, University of Murcia, Murcia, Spain; 3 Department of Physiology, School of Medicine, University of Murcia, Murcia, Spain; 4 Preclinical Imaging Unit, Laboratory Animal Service, University of Murcia, Murcia, Spain; 5 GENZ-Group of Research on Enzymology, Department of Biochemistry and Molecular Biology-A, Regional Campus of International Excellence "Campus Mare Nostrum", University of Murcia, Murcia, Spain; University of Montreal, CANADA

## Abstract

**Objective:**

The aim of this study was to compare *in vivo vs ex vivo* liver stiffness in rats with acoustic radiation force impulse (ARFI) elastography using the histological findings as the gold standard.

**Methods:**

Eighteen male Wistar rats aged 16–18 months were divided into a control group (n = 6) and obese group (n = 12). Liver stiffness was measured with shear wave velocity (SWV) using the ARFI technique both *in vivo* and *ex vivo*. The degree of fibrosis, steatosis and liver inflammation was evaluated in the histological findings. Pearson’s correlation coefficient was applied to relate the SWV values to the histological parameters.

**Results:**

The SWV values acquired in the *ex vivo* study were significantly lower than those obtained *in vivo* (*P* < 0.004). A significantly higher correlation value between the degree of liver fibrosis and the ARFI elastography assessment was observed in the *ex vivo* study (r = 0.706, *P* < 0.002), than the *in vivo* study (r = 0.623, *P* < 0.05).

**Conclusion:**

Assessment of liver stiffness using ARFI elastography yielded a significant correlation between SWV and liver fibrosis in both the *in vivo* and *ex vivo* experiments. We consider that by minimising the influence of possible sources of artefact we could improve the accuracy of the measurements acquired with ARFI.

## Introduction

Non-alcoholic fatty liver disease (NAFLD) is one of the most common causes of chronic hepatopathy in adults, with a prevalence of up to 20–30% in developed countries [1]. This entity represents a wide spectrum of pathologies ranging from a simple steatosis (80–90% of cases) to steatohepatitis (10–20%) [2]. Moreover, in the absence of diagnosis and early treatment it can lead to a progressive liver fibrosis and subsequent liver cirrhosis [3].

Today’s gold standard for assessing liver involvement is still biopsy. However, it does have considerable limitations and complications, some of which, though infrequent, are potentially fatal [4,5]. This makes it necessary to find a non-invasive diagnostic method to enable an accurate and reproducible assessment of this pathology.

The acoustic radiation force impulse (ARFI) elastography technique, capable of evaluating stiffness of the liver parenchyma through short-duration high-intensity acoustic pulses, has yielded promising results, which makes it an important diagnostic alternative for NAFLD assessment, even with normal laboratory values [6]. Various studies [7–10] have focused on the assessment of liver fibrosis and the possible degree of influence that hepatic steatosis and associated inflammatory processes may have on the final result [11–16]. However, a greater number of experiments are needed to focus on external factors that may influence the acquisition of measurements. Bruno et al [17] claim there are physical, geometrical, anatomical and physiological factors that influence shear wave velocity (SWV) measured with ARFI. Characteristics such as movement during the acquisition of measurements [18], the depth of the region of interest (ROI) [19–21], the ultrasound frequency used [20–23], the extrinsic compression exerted by the transducer [24], the orientation of the ROI with regard to the surface of the target organ [21] and physiological factors such as heartbeat, respiratory movements or fasting [20, 25], might alter the assessment of liver stiffness. Our hypothesis is that these factors could considerably alter the accuracy of ARFI velocities.

The aim of this study was to compare *in vivo* and *ex vivo* liver stiffness in rats with the ARFI technique using the histological findings as the gold standard.

## Materials and methods

### Animal study

A total of 18 Wistar male rats aged 16–18 months were maintained under constant cycles of light-dark (12:12 hours) and temperature (25°C) in the Murcia University animal-housing unit. They were divided into two groups: control group (n = 6) and obese group (n = 12). One of the animals died during the *in vivo* experiment and was excluded from the study. All the rats were fed a diet made up of 61% carbohydrates, 15% fats and 24% proteins. Subsequently, the obese group was administered a diet with a high fat content (Harlan TD.06414) during the 6 weeks prior to the study, in which 60.3% of the kcals came from fat (37% saturated; 47% monounsaturated; 16% polyunsaturated) and the rest from carbohydrates (21.3%) and proteins (18.4%).

Before ultrasound study, animals were heavily sedated with an intraperitoneal injection of 0.1 ml/100 gr sodium pentobarbital in order to perform an *in vivo* ultrasound evaluation. The same drug caused the animals death 10–15 minutes after the injection and the *ex vivo* ultrasound study and blood and liver extraction were performed.

### Ethics statement

The experimental protocols were designed according to the “Guiding principles for research involving animals and humans” adopted by the American Physiology Society and the European Union standards and received approval from the University of Murcia’s “Institutional Animal Care and Use Committee” (CEEA) in process number A1320140709.

### Laboratory tests

A blood extraction (5 ml) was taken by cardiac puncture. The blood was anticoagulated with 0.1 ml of EDTA and kept cold until centrifugation (3000 rpm for 15 minutes). The plasma samples were stored in Eppendorf Tubes at -80°C until the measurements were taken. A complete lipid profile was obtained, with calculation of HDL (mg/dl), LDL (mg/dl) and triglyceride levels (mg/dl) using an ELISA Kit from “Shanghai Yehua Biological Technology” (Shanghai, China). We also calculated the levels of alanine aminotransferase (ALT) (U/ml) using a kit supplied by Cayman Chemical (ref. 700260) for laboratory assessment of liver damage.

### Protocol for the ARFI technique

**Fig 1:**
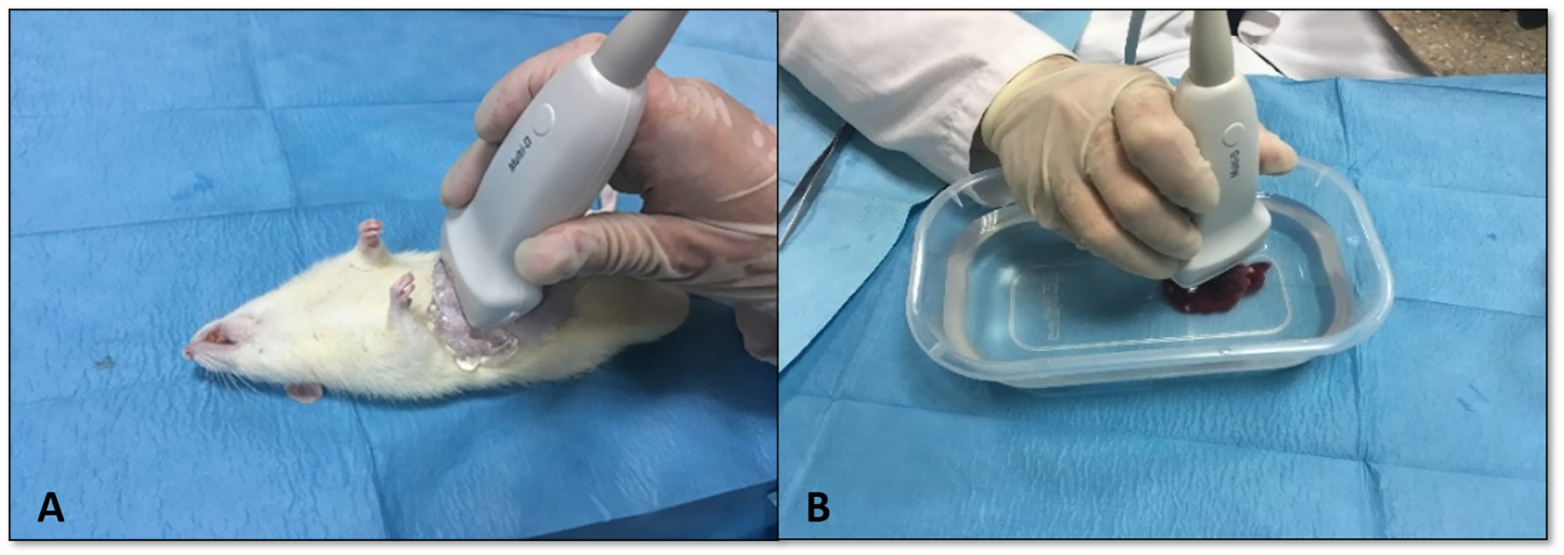
**A**, *In vivo* ultrasound study with the animal placed in decubitus. **B**, *Ex vivo* ultrasound study using the immersion technique.

All the ultrasound studies were performed by a radiologist with more than 20 years’ experience in conventional ultrasonography and more than 10 years with the ARFI technique, who was blind to the laboratory and histology results. The ARFI values were obtained with an Acuson S2000 system (Siemens, Erlangen, Germany) using the Virtual Touch Tissue Quantification software, which is capable of generating and detecting the transverse or shearing waves used to determine ARFI values, represented as SWV measured in metres per second. A 9L4 transducer was used, with a 700 cycle push transmit event at either 4,44 or 5,71MHz, depending on the region of interest (ROI) depth, and a shear wave bandwidth of 123-158Hz. Fig 1 shows both the conventional ultrasound study and the ARFI elastography measurements in the liver parenchyma, which were taken both *in vivo*, with the animals anaesthetised (Fig 1A), and *ex vivo*, after they were euthanised, with the liver explant placed in a plastic container with physiological serum for an immersion ultrasound study to be conducted (Fig 1B) [26].

To determine the ARFI values we established three ROIs in the animals’ right liver lobes (medial and lateral) in both the *in vivo* and *ex vivo* study. Five measurements were taken in each selected ROI. These regions had been studied previously by conventional ultrasound. The ROIs were situated at a depth of 1-2cm in the liver parenchyma, away from the capsular region and major intrahepatic vascular structures and including the largest possible amount of liver parenchyma; a minimum extrinsic compression was exerted with the transducer on the abdominal wall in the *in vivo* study with application of physiological serum as a connector; the ROIs were arranged parallel to the capsular surface and five measurements were obtained for each ROI to avoid error variability. The ARFI result in each ROI, expressed as SWV measured in metres/second, was expressed as the mean and standard deviation of the five measurements obtained for each one.

### *In vivo* ARFI

**Fig 2:**
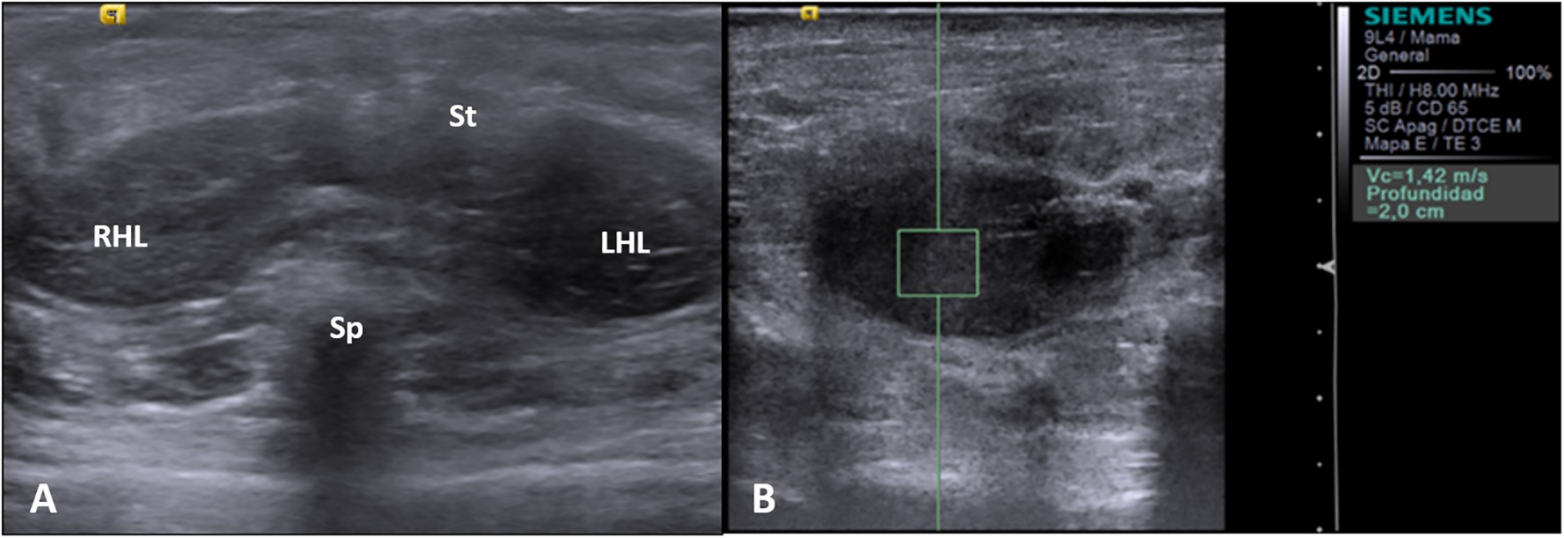
**A**, *In vivo* ultrasound image of the upper abdominal quadrants. RHL: right hepatic lobe; LHL: left hepatic lobe; St: Stomach; Sp: Spine. **B**, *In vivo* assessment using the ARFI technique.

For the *in vivo* ultrasound study the animals’ abdominal fur was removed completely using a depilatory cream to avoid the formation of artefacts. The animals were then heavily sedated to induce a drop in respiratory and heart rate in order to reduce the artefacts caused by the heartbeat and movements in the thoracic cavity. Immediately after injection of the drug the animals were placed and secured in a decubitus position to enable adequate ultrasound exploration. For the B-mode ultrasound study of the liver parenchyma we explored the upper abdominal quadrants, focusing on the subcostal region, until we located the two right liver lobes. During the conventional study we explored the right liver lobe thoroughly and established the ROIs that would subsequently be used for recording the SWVs, as shown in Fig 2.

### *Ex vivo* ARFI

**Fig 3:**
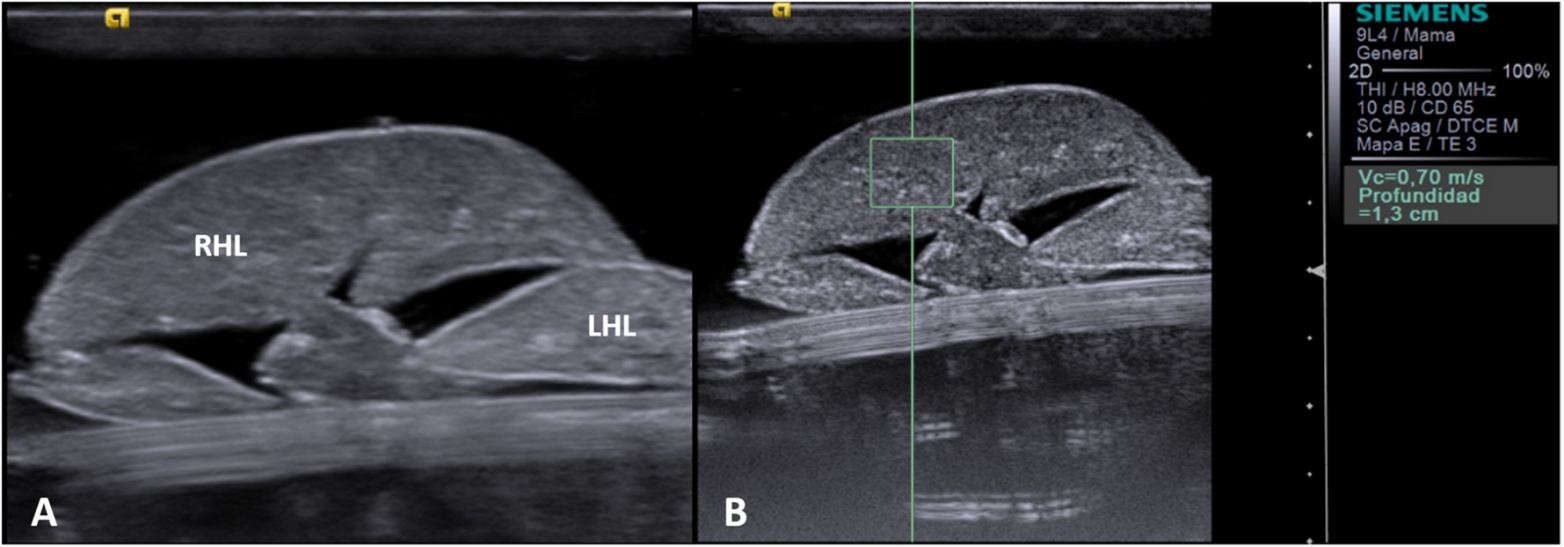
**A**, *Ex vivo* ultrasound image of the liver explant. RHL: right hepatic lobe; LHL: left hepatic lobe. **B**, *Ex vivo* assessment using ARFI.

Once the animals had been euthanised their liver parenchyma was removed for the *ex vivo* ultrasound study. The explants were placed in a jar with physiological serum before the study. We then introduced the liver explant into a plastic container with physiological serum at room temperature to conduct an immersion ultrasound study. As in the *in vivo* study, the two right liver lobes were located to establish the ROIs, as shown in Fig 3.

### Histology

The liver samples were placed in histology cassettes (Labolan, Navarra, Spain) and submerged in 4% commercial formalin buffered in PBS (pH 7.0, Panreac Química, Barcelona, Spain) for subsequent processing using a multifunctional microwave histoprocessor (Milestone KOS Histostation, Milestone, Bergamo, Italy) and inclusion in paraffin. The histological analysis was conducted on the liver samples with haematoxylin-eosin (HE) and Masson trichrome (TRIC) staining for assessment of steatosis, inflammatory processes and liver fibrosis. The inflammatory infiltrate (CD3+ T lymphocytes) was also determined by immunohistochemical determination on liver sections in paraffin using an indirect colorimetric technique based on the avidin-biotin-peroxidase complex (ABC technique).

The fibrosis surface area was calculated quantitatively and semi-quantitatively. The quantitative assessment was done by histomorphometric analysis using a specific software package (AxioVision Rel. 4.8, Zeiss); the connective tissue surface was calculated in 10 random fields from the liver sections of each animal and the result expressed as mean standard deviation of the fibrosis surface of the 10 fields. In addition, the semi-quantitative METAVIR scale was used to assess the liver fibrosis [27]. This scale is divided into five stages: F0, no fibrosis; F1, perisinusoidal or periportal fibrosis; F2, perisinusoidal and periportal fibrosis; F3, fibrous bridges; F4, cirrhosis.

Positive immunoreaction (CD3+ T lymphocytes) was identified as a dark brown pericellular halo. To establish possible differences in CD3+ T lymphocyte infiltration depending on the technique, we calculated the weighted mean ± standard deviation of the positive cell count in a minimum of 10 random fields at high magnification (x400) of each animal’s liver sections. To determine the grade of steatosis we used the scale defined by Brunt et al [28] based on the percentage of cellular lipid overload: grade E0, no steatosis; grade E1, 0–33%; grade E2, 33–66%; grade E3, >66%. The histological and immunohistochemical studies were performed by a veterinary anatomical pathologist with 7 years’ experience.

### Statistical analysis

The statistical analysis was carried out using the SPSS software package version 15.0 (SPSS for Windows, Chicago, IL, USA). The Kolmogorov-Smirnov test was used to verify that the quantitative variables followed a normal distribution. In this way, parametric statistical tests were applied due to their greater statistical power.

The Student t test was applied to determine differences between the intergroupal means of the quantitative variables (surface area of liver fibrosis per field, number of hepatic CD3 lymphocytes per field and SWVs obtained with ARFI in the liver parenchyma) and to establish differences between quantitative variables and dichotomous variables (Brunt classification [27]). To analyse the degree of correlation between the SWVs and the quantitative histological variables (surface area of liver fibrosis per field, number of CD3 lymphocytes in the liver parenchyma per field and percentage of steatosis) we applied Pearson’s correlation coefficient. Statistical significance was defined as *P* < 0.05.

## Results

### Laboratory parameters

The HDL, LDL and triglyceride values showed no significant differences between the animals in the control group and obese group. Likewise, the ALT values showed no significant differences between the two groups.

### ARFI determinations and correlation analysis

**Fig 4:**
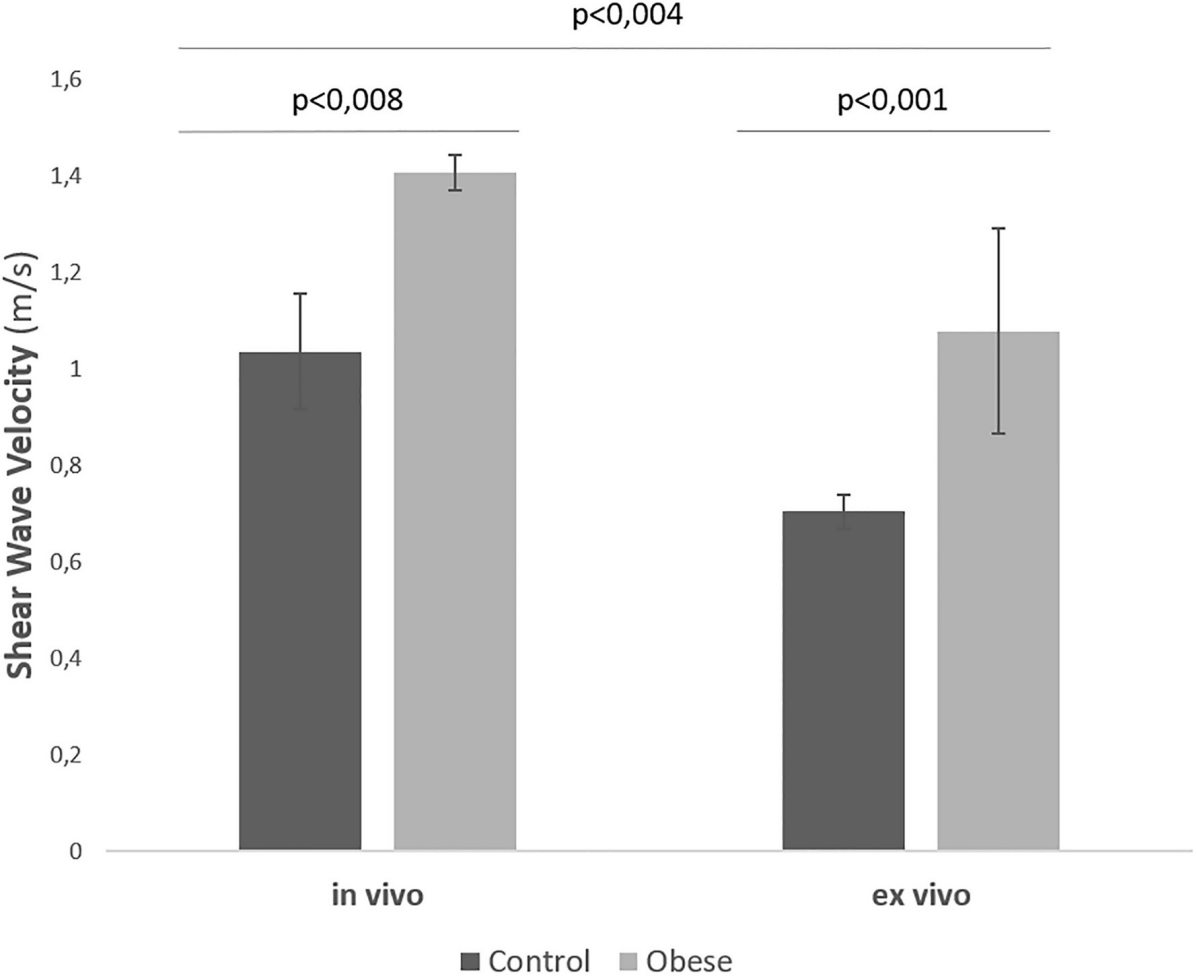
Mean comparative values of SWV with ARFI in the liver parenchyma *in vivo* and *ex vivo* in the control and obese group.

The SWVs obtained in the liver parenchymas of the control group gave an *in vivo* mean of 1 ± 0.2 m/s whereas the obese group showed a higher mean in the *in vivo* assessment (1.4 ± 0.1 m/s), with a statistically significant difference (*P* < 0.008). As for the *ex vivo* explorations the SWVs obtained were 0.7 ± 0.1 m/s in the control group and a significantly higher mean of 1.1 ± 0.2 m/s in the animals from the obese group (*P* < 0.001). Fig 4 shows a clear increase in SWV in the *in vivo* determinations compared to the *ex vivo* study, in both the control group and obese group (*P* < 0.004).

With regard to the correlation for the quantitative histological liver variables, the whole study sample showed a positive and statistically significant correlation between the mean SWV obtained by ARFI and the surface area of liver fibrosis per field, in both the *in vivo* study (r = 0.623, *P* < 0.008) and *ex vivo* study (r = 0.706, *P* < 0.002), the *ex vivo* value being higher than the *in vivo* value. The correlation between the SWV with ARFI and the number of CD3+ T lymphocytes was positive and statistically significant in the *in vivo* study (r = 0.670, *P* < 0.003), but not statistically significant in the *ex vivo* study (r = 0.471, *P* < 0.056).

If we observe the degree of correlation between the SWV with ARFI and the grade of liver steatosis we see a positive but not statistically significant correlation in the *ex vivo* study (r = 0.530, *P* = 0.029) and an absence of correlation in the *in vivo* study (r = 0.120, *P* < 0.647).

### Histological findings

**Fig 5:**
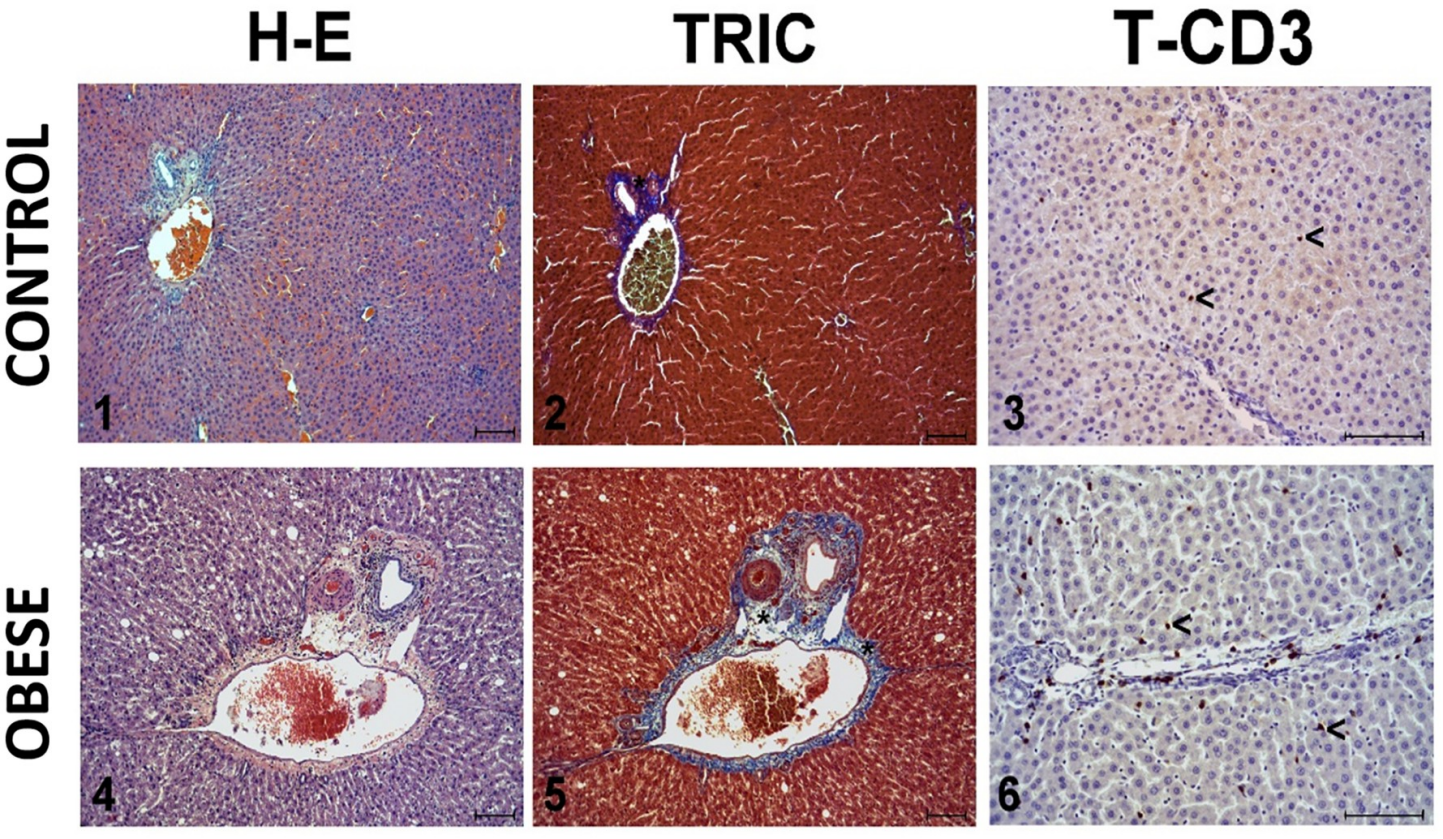
Representative images of the liver histopathology examination (1 & 4), surface area of fibrosis (2 & 5, asterisks), and CD3 T lymphocyte infiltration (3 & 6, arrowheads) in the control group (1–3) and obese group (4–6). Haematoxylin-eosin technique (HE) (1 & 4), Masson’s trichrome staining (TRIC) (2 & 5) and ABC anti-CD3 T lymphocytes (T-CD3) (3 & 6). Scale: 100 micrometres.

**Fig 6:**
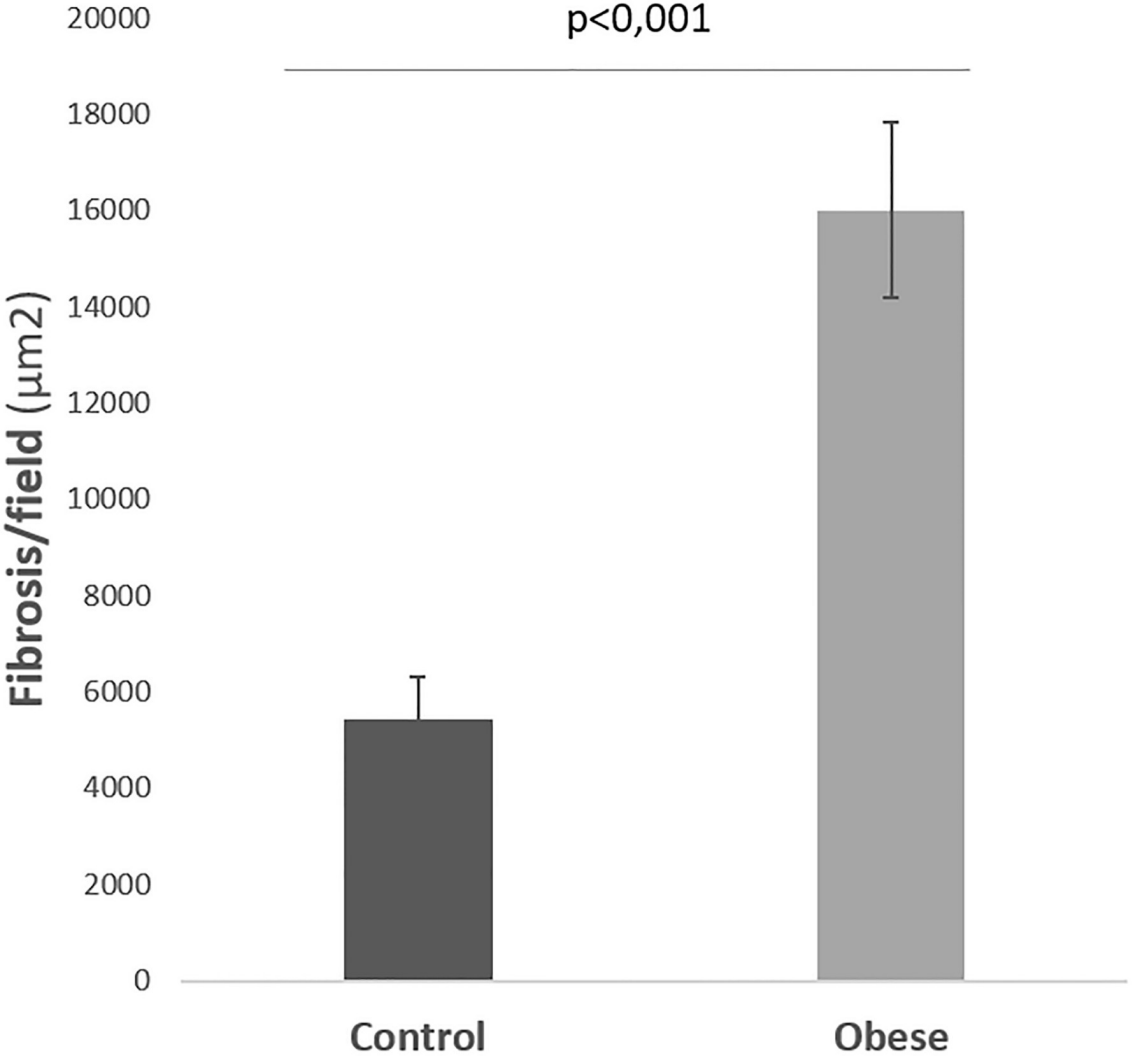
Mean surface area of liver fibrosis in the control group and obese group.

**Fig 7:**
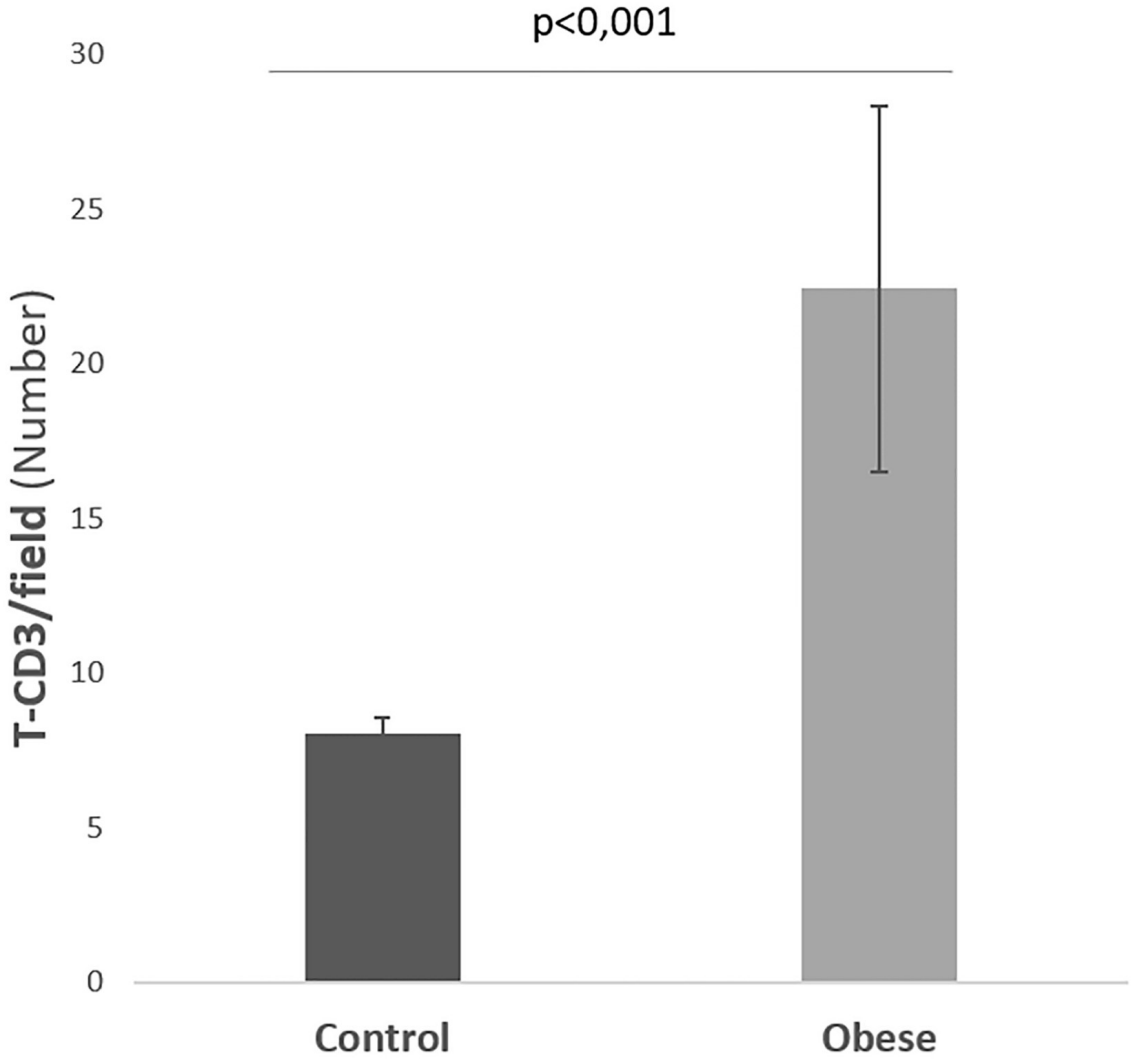
Mean value of the CD3^+^ T lymphocyte count in the liver parenchyma in the control group and obese group.

Fig 5 shows the histological findings. Fig 6 shows that the mean surface area of liver fibrosis per field in the control group animals was 5423 ± 893 μm^2^, a result in contrast to the mean of 16006 ± 1818 μm^2^ obtained in the obese group, with statistically significant differences (*P* < 0.001). According to the METAVIR score the control group animals had no significant fibrosis (F0) whereas the obese group animals developed a mild fibrosis (F1). Fig 7 shows the degree of CD3+ T lymphocyte infiltration: the control group had a count of 8.1 ± 0.6 lymphocytes/field whereas the obese group showed a clearly higher value (22.4 ± 5.9 lymphocytes/field) (*P* < 0.001).

**Fig 8:**
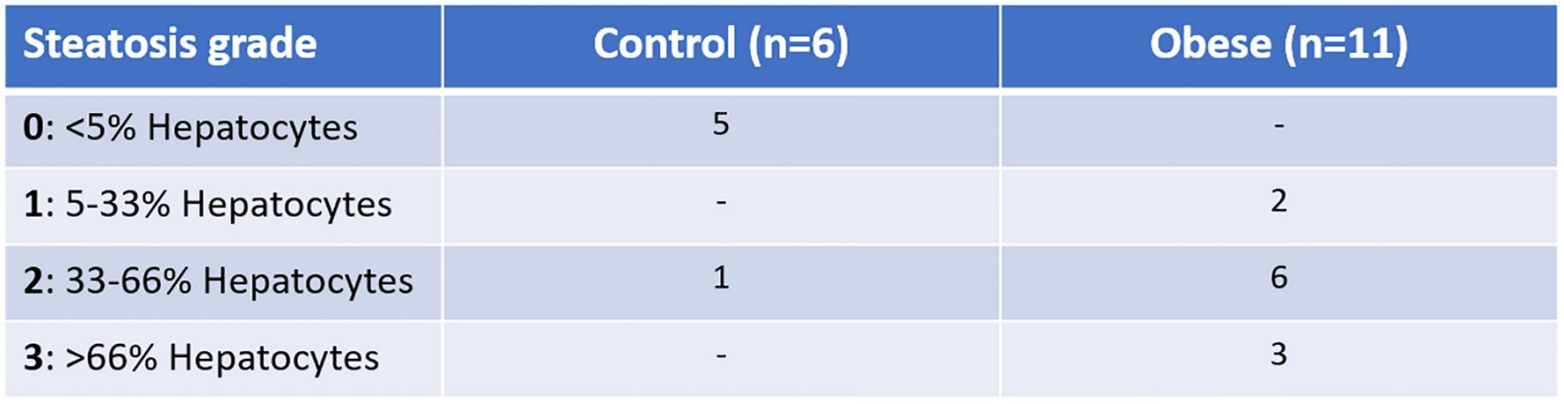
Steatosis grade in the control group and obese group.

Analysis of the degree of liver steatosis revealed that the control group animals did not show significant steatosis except one of the rats with a moderate lipid overload (E0 = 5; E2 = 1), whereas the obese group animals presented a significant increase in steatosis (*P* < 0.002), mostly with a medium or severe degree of fat overload (E1 = 2; E2 = 6; E3 = 3) (Fig 8).

## Discussion

The present study is a comparative analysis between liver parenchyma assessment using the ARFI technique in *in vivo* experiments, with the animals placed in the supine position and under heavy sedation to avoid any possible cardiac or respiratory artefacts, and in *ex vivo* experiments, after euthanisation of the animals and with the liver explants introduced into a container with physiological serum to minimise possible sources of artefact, such as those related to physiological factors (breathing, heartbeat), and improve the conditions in which we took the measurements.

Several factors have been described as capable of distorting SWVs acquired with ARFI. According to publications by Bruno et al [17] when performing explorations with the ARFI technique the radiologist must take into consideration all these physical, geometrical, anatomical and physiological factors that can alter the measurements and lead to diagnostic errors of interpretation and possible wrong treatment. One of the most relevant is the depth at which the measurements are established. This factor particularly influences the study of voluminous organs like the liver. Independent studies conducted by D’Onofrio, Gallotti & Mucelli [19] and Kamimuma et al [20] show significant differences according to the depth at which the ROI is established in the liver parenchyma and observe that the deeper the ROI in the parenchyma the lower the SWVs. Furthermore, the study by Chang, Kim, Kim & Lee [21] reports a considerable increase in the variability of measurements according to the depth of the ROI; they establish an ideal ROI depth of 2-3cm below the liver capsule using high-frequency probes, and 4-5cm when using low-frequency probes, in order to minimise dispersion in the results. Due to the small liver size in our study we selected a ROI including just the liver parenchyma and avoiding the liver capsule and large intrahepatic structures.

Other factors reported in the literature, as the ultrasound frequencies used to determine the SWVs or the frequency bandwidth of generated shear waves, are controversial. The study developed by Chang et al [21] claims that SWVs acquired with a low frequency probe had a tendency to be higher at the same depth, while Dillman et al [22] found no significant differences between low and high frequency probes. Another study published by Kazemirad et al [23] assessed liver shear stiffness using US elastography at low (40-130Hz) and high (130-220Hz) frequencies, obtaining better distinction of steatohepatitis categories at high frequencies. Throughout our experiment we used a 9L4 probe at either 4,44 or 5,71MHz, depending on the ROI depth, at high frequencies (123–158 Hz) to determine the SWVs.

Regarding the influence of the extrinsic compression of the tissue with the transducer, there are several studies such as that by Syversveen et al [24] which show significant changes in measurement depending on the degree of compression exerted, in this case in studies on the kidney parenchyma. In the *in vivo* model we exert minimum compression on the animal’s abdominal wall to achieve a good exploratory window. In the *ex vivo* study we minimise this factor because the good transmission of the ultrasound beam through the serum used for the liver study in our experimental model enables us to avoid excessive compression for the collection of data.

The arrangement of the ROIs parallel to the liver capsule has also proved relevant in determining ARFI values. Authors such as Chang et al [21] emphasise this factor as a way of avoiding variability between measurements because the ultrasound beams interact with a larger number of interfaces and collect a greater amount of information. The possibilities of manipulating the position of the organs in the *ex vivo* studies made it easier to correctly align the ROIs. The influence of physiological factors, like heartbeat, respiratory movements or fasting, has also been reported on the accuracy of SWV measurements. The study published by Kamimuma et al [20], which analyses the liver parenchyma using ARFI, claims that the acquisition of measurements during deep breathing or following the ingestion of food does not distort the measurements, whereas other publications such as the study by Mederacke et al [25] report significant differences between the fasting and post-ingestion states. In our *ex vivo* model we limited all the potential sources of physiological artefacts that might affect liver stiffness measurements.

After conducting all the explorations in the two measurement models we observed that the mean SWV obtained in the liver parenchyma in the *in vivo* studies was significantly higher than that acquired in the *ex vivo* studies. Inclusion of the liver explants in a homogeneous medium, without artefacts caused by physiological processes and with the possibility of correctly aligning the organs to establish the ROIs in an optimal way, appears to reduce the SWVs in the ARFI assessment. Minimising the number of factors that can alter the measurements would make these measurements more accurate and closer to real parenchymal elasticity. These findings suggest that in addition to physiological artefacts and those related to measuring correctly, the abdominal cavity and adjacent structures might be factors that alter measurements. This might be interesting when assessing grades of mild and moderate fibrosis, where the ARFI technique shows considerable limitations due to the major overlapping of results [29].

The ARFI technique has yielded promising results to establish itself as an important non-invasive diagnostic alternative for assessing NAFLD, even with normal laboratory values. With our Wistar rat model we found no significant differences between the control group and obese group for the results of the lipid profile or ALT levels, but we did obtain differences between the control animals and obese animals when using ARFI, which shows an important correlation with histology. This is especially relevant, as the ARFI technique could give us early identification of histological alterations, particularly in less advanced stages of NAFLD, where the laboratory parameters are normal. Various studies, such as that by Guzmán-Aroca et al [6], have obtained similar results in patients who, without presenting significant laboratory abnormalities, did show histopathological alterations in the liver. We cannot therefore exclude a diagnosis of NAFLD and histological alterations in patients who do not present laboratory abnormalities. In these patients the use of ARFI elastography may be differential when establishing suitable management to prevent the disease from evolving. Moreover, ARFI has shown very promising results for detecting and grading liver fibrosis in different stages [7, 30]; this is the histological feature best studied by elastography and an important prognostic factor in the development of NAFLD. Our study revealed an important correlation between SWV and the grade of liver fibrosis according to the affected surface, in both the in *vivo* and *ex vivo* studies. This shows that by minimising possible sources of artefact for the detection and quantification of SWVs we obtain more reliable results for characterising liver fibrosis.

Another of the aims of the ARFI technique is based on detecting liver fibrosis at early stages of the disease, when the fibrogenic process is in its initial stages, in order to establish means to prevent it from progressing. Some studies, such as that by Bota et al [7], claim that ARFI is capable of diagnosing F≥2 liver fibrosis stages with a sensitivity of 74% and specificity of 83%, both of them rising to 87% when identifying F4. These results highlight the capacity of the ARFI technique for differentiating moderate and advanced stages of fibrosis from stages with non-significant or mild fibrosis. Another study, by Sporea et al [29], shows an important superposition of results in patients without fibrosis (F0) and patients with mild (F1) or moderate (F2) fibrosis. Although the grade of fibrosis developed by the animals in our study was quite low, we found significant differences between those that showed no significant liver fibrosis (F0 on the METAVIR scale) and those presenting with some degree of fibrosis (F≥1), in both the in *vivo* and *ex vivo* studies. These results indicate that by adjusting the parameters correctly and optimising SWV measurement the ARFI technique might be a fundamental tool for differentiating healthy patients from those with an early stage of liver involvement.

As for the capacity of ARFI to characterise hepatic steatosis and necroinflammatory processes, histological features that are also present in NAFLD and chronic hepatopathy of various origins, there is a greater degree of controversy in the literature. Fierbinteanu-Braticevici et al [8] revealed in their research that the ARFI values presented an important negative correlation with the grade of hepatic steatosis, with a progressive reduction in SWVs as the grade of steatosis increased. However, another study by Nishikawa et al [31] found no significant correlation between the SWVs acquired by ARFI and the degree of steatosis. We found no significant correlation in our animal model between the degree of lipid overload and the SWVs, in either the *in vivo* or *ex vivo* study, but we did detect significant differences between animals without significant lipid overload on the Brunt scale [28] (E0) and animals with some degree of steatosis (E ≥ 1) in both the *in vivo* and *ex vivo* studies. Our results suggest therefore that the ARFI technique could be useful for differentiating the absence of lipid overload from the other grades of hepatic steatosis, from mild to severe following a proper optimisation of SWV acquisition.

As occurred in the case of steatosis, there is a certain degree of controversy in the assessment of necroinflammatory processes. The studies conducted by Fierbinteanu Batricevici et al. [8] showed a certain degree of positive correlation with the degree of inflammation. Conversely, Yoneda et al [32] reported SWV differences between groups with different degrees of inflammation but did not identify a gradual change in SWVs between the different degrees of inflammation. We only found a significant positive correlation in our experiment between the SWV values determined *in vivo* and the number of hepatic CD3 T lymphocytes obtained in the histological study; however, we were unable to detect a correlation in the *ex vivo* study. Further studies with the ARFI technique are necessary to see if it is useful in detecting and grading steatosis and liver inflammation in patients with NAFLD. An improved selection of ARFI parameters and ROI location, as in the case of fibrosis, might increase the accuracy of the technique in assessing fatty liver overload and degree of inflammation.

### Limitations and future research

In our *ex vivo* study we cannot exclude the possible influence of physiological serum on the liver explant when assessing liver stiffness, nor can we rule out that the measurements are altered by the absence of hydrostatic blood pressure. It would be interesting in future research to obtain a model in which the acquisition of measurements depends solely on liver stiffness. We could therefore calculate a correction factor to try to adjust the values obtained *in vivo*. Moreover, it was not easy to perform the ARFI examination due to the small liver size and the fixed dimensions of the ROI. However, the SWV measurements were taken adequately. It is also worth noting that to avoid interobserver variability the ARFI technique was performed by an examiner with experience. However, there is shown to be an excellent interobserver agreement with the ARFI technique [33].

## Conclusions

This is the first experimental study to determine SWVs in the liver with the ARFI technique both *in vivo* and *ex vivo*. The SWVs are seen to be higher *in vivo* than those measured *ex vivo*. This suggests that various factors may alter the liver stiffness measurements. Furthermore, we detected some very high correlation values between SWV and the degree of liver fibrosis in both the *in vivo* and *ex vivo* experiments. In addition, a greater degree of correlation was identified in the *ex vivo* study, which confirms that by planning and optimising the determinations with ARFI to limit sources of artefact we can obtain more reliable, more accurate and more reproducible results. We consider that further studies are needed to clarify the possible role of ARFI in this issue.

## Supporting information

S1 Table Animal group and quantitative and semiquantitative variables. (XLSX)
